# MicroRNAs as regulators of cardiac dysfunction in sepsis: pathogenesis and diagnostic potential

**DOI:** 10.3389/fcvm.2025.1517323

**Published:** 2025-02-18

**Authors:** Zhen Liu, Feiyang Li, Ningcen Li, Yong Chen, Zelin Chen

**Affiliations:** ^1^ Research Center of Experimental Acupuncture Science, Tianjin University of Traditional Chinese Medicine, Tianjin, China; ^2^ School of Acupuncture-Moxibustion and Tuina, Tianjin University of Traditional Chinese Medicine, Tianjin, China; ^3^ Department of Critical Care Medicine, Tianjin Hospital of ITCWM Nankai Hospital, Tianjin, China; ^4^ National Clinical Research Center for Chinese Medicine Acupuncture and Moxibustion, Tianjin, China

**Keywords:** microRNAs, sepsis-induced cardiac dysfunction, progression, diagnosis, mechanisms

## Abstract

**Introduction:**

Sepsis, a life-threatening condition arising from an uncontrolled immune response to infection, can lead to organ dysfunction, with severe inflammation potentially causing multiple organ failures. Sepsis-induced cardiac dysfunction (SIMD) is a common and severe complication of sepsis, significantly increasing patient mortality. Understanding the pathogenesis of SIMD is crucial for improving treatment, and microRNAs (miRNAs) have emerged as important regulators in this process.

**Methods:**

A comprehensive literature search was conducted in PubMed, Science Direct, and Embase databases up to September 2024. The search terms included [“miRNA” or “microRNA”] and [“Cardiac” or “Heart”] and [“Sepsis” or “Septic”], with the language limited to English. After initial filtering by the database search engine, Excel software was used to further screen references. Duplicate articles, those without abstracts or full texts, and review/meta-analyses or non-English articles were excluded. Finally, 106 relevant research articles were included for data extraction and analysis.

**Results:**

The pathogenesis of SIMD is complex and involves mitochondrial dysfunction, oxidative stress, cardiomyocyte apoptosis and pyroptosis, dysregulation of myocardial calcium homeostasis, myocardial inhibitory factors, autonomic nervous regulation disorders, hemodynamic changes, and myocardial structural alterations. miRNAs play diverse roles in SIMD. They are involved in regulating the above-mentioned pathological processes.

**Discussion:**

Although significant progress has been made in understanding the role of miRNAs in SIMD, there are still challenges. Some studies on the pathogenesis of SIMD have limitations such as small sample sizes and failure to account for confounding factors. Research on miRNAs also faces issues like inconsistent measurement techniques and unclear miRNA-target gene relationships. Moreover, the translation of miRNA-based research into clinical applications is hindered by problems related to miRNA stability, delivery mechanisms, off-target effects, and long-term safety. In conclusion, miRNAs play a significant role in the pathogenesis of SIMD and have potential as diagnostic biomarkers. Further research is needed to overcome existing challenges and fully exploit the potential of miRNAs in the diagnosis and treatment of SIMD.

## 1 Introduction

Sepsis is a life-threatening condition characterized by organ dysfunction caused by a dysregulated response to infection. Severe inflammatory reactions can lead to multiple organ failures, significantly threatening human health (1–5). In 2017, the World Health Organization (WHO) reported that nearly 30 million people worldwide suffer from sepsis yearly, with a mortality rate of approximately 25%–30% (6). Sepsis-induced cardiac dysfunction (SIMD) is a common complication of severe sepsis, with about 50% of patients experiencing some degree of myocardial injury (7). When sepsis progresses to cardiac dysfunction, the patient mortality rate increases 3–4 times (8) to reach about 80% (9), seriously threatening the lives of patients. Although several studies have been conducted on SIMD, the pathogenesis of SIMD is complex and not yet fully understood, resulting in slow progress in its treatment and a lack of specific treatment plans. In addition, the high cost and consumption of medical resources significantly impact the quality of life of patients. Recent studies have shown that epigenetic modifications such as microRNAs (miRNAs) play a critical role in the progression (10), diagnosis, and treatment of SIMD and are considered a significant breakthrough in understanding the pathophysiological mechanisms of SIMD.

Epigenetic modifications refer to the genetic phenomenon of altering deoxyribonucleic acid (DNA) and chromatin structures through chemical changes that affect gene expression (11). Common epigenetic modifications include: (1) DNA methylation, changing the gene expression pattern by adding a methyl group to the DNA molecules; (2) Histone modifications that alter chromatin structure and density, such as acetylation, acylation, methylation, and ubiquitination; (3) Chromatin remodeling, including histone replacement, transfer, and rearrangement of histone; (4) Recognition and linking of histones and DNA, involving the regulation and mediation of histone and DNA binding, such as histone modification readers; and (5) Non-coding RNAs, including miRNAs and long non-coding RNAs. These epigenetic modifications interact with each other and collectively affect gene expression and cell fate. Epigenetic modifications are critical in many biological processes, such as development, cell differentiation, and disease occurrence. MiRNAs are a class of non-coding RNAs, 19–24 nucleotides in length, characterized by a “hairpin loop” structure. It is mainly involved in post-transcriptional regulation through specific binding to the 3'-Untranslated Region (3'-UTR) of the target gene. It has been reported (12) that miRNAs play a key role in maintaining physiological homeostasis, regulating growth and development, and disease progression. Many types of miRNAs regulate various pathophysiological processes in the body, especially inflammatory cardiovascular diseases, and may play an important role in SIMD. Studies have shown that miRNAs are involved in the pathogenesis of SIMD, making them potential biomarkers for SIMD diagnosis and treatment. This review explores the role and mechanism of miRNAs in SIMD to clarify the potential value of miRNAs as a biomarker and diagnosis of SIMD. The review also provides a new direction for the application of miRNAs in diagnosing and treating SIMD.

## 2 Method

### 2.1 Search strategy

We searched PubMed, Science Direct, and Embase databases for studies published from inception through September 2024. The search keywords employed were as follows: [“miRNA” or “microRNA”] and [“Cardiac” or “Heart”] and [“Sepsis” or “Septic”]. The search was as follows: ((((“miRNA”) OR (“microRNA”)) OR (“MicroRNAs"[Mesh])) AND (((“Heart”) OR (“Cardiac”)) OR (“Heart"[Mesh]))) AND (((“Sepsis”) OR (“Septic”)) OR (“ Sepsis” [Mesh])). The language was limited to English. The database search engine was used to perform the initial filtering, which identified 269 relevant articles.

### 2.2 Study selection

**Figure 1:**
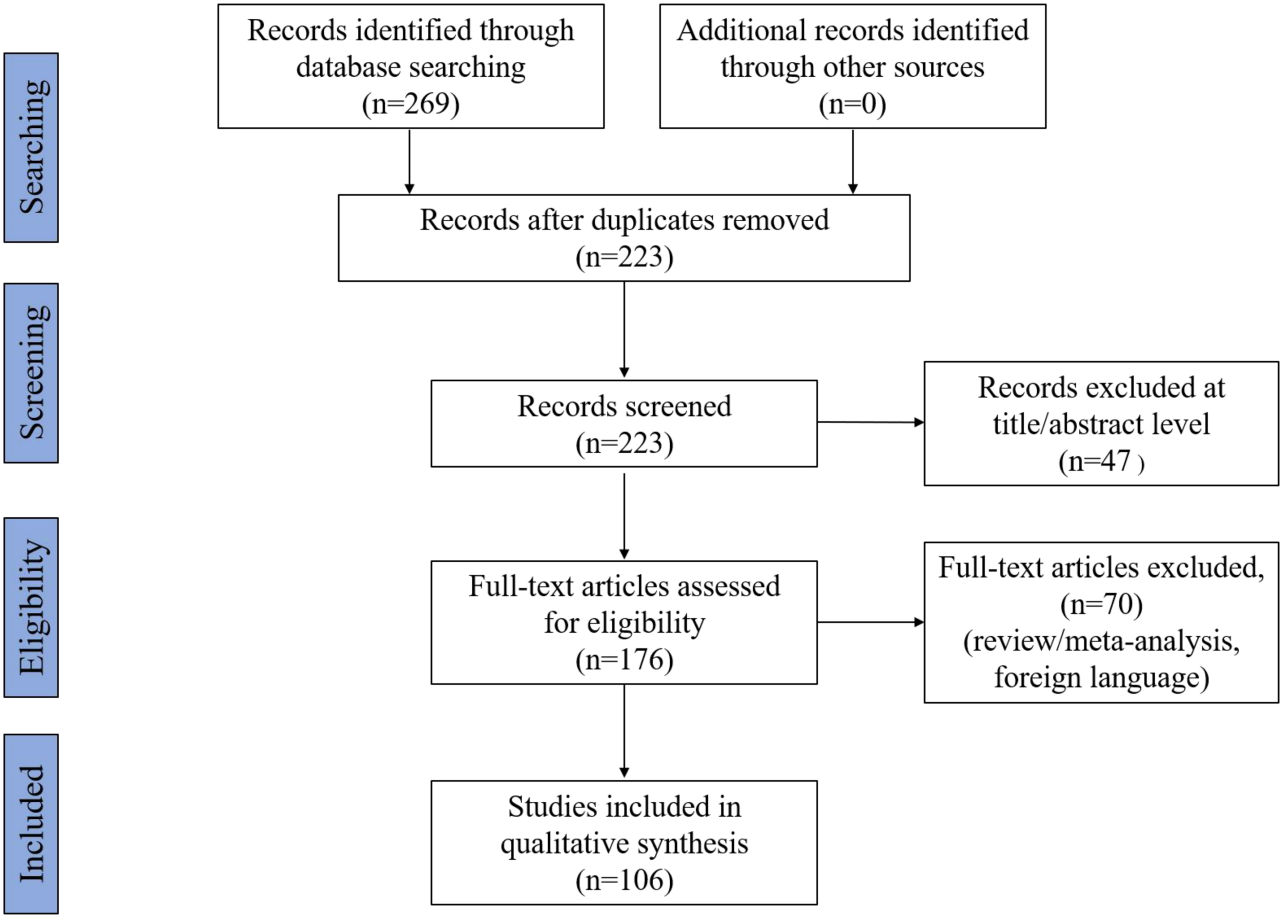
Flowchart of the search strategy and process.

Before reading the full text intensively, we used Excel software to select references that matched the topic. We excluded 46 duplicate articles, 47 without abstract or full text, and 70 review/meta-analyses or non-English articles. Finally, 106 full texts of research articles that met the inclusion criteria were included. A flow chart of the search process is shown in Figure 1.

### 2.3 Data extraction

In the mechanistic study of cardiac dysfunction in sepsis, the design data were extracted and classified using a pre-defined data extraction table, indicating the model type, miRNAs, target gene, function, and outcome measures (SIMD-related behavior and indicators of mechanism). Data were extracted by one author and reviewed by other authors. Due to the similarity of some studies, the table lists information from partially or fully representative and recently published studies to analyze the mechanisms involved in miRNAs in SIMD.

## 3 Results

### 3.1 Pathogenesis of SIMD

**Figure 2:**
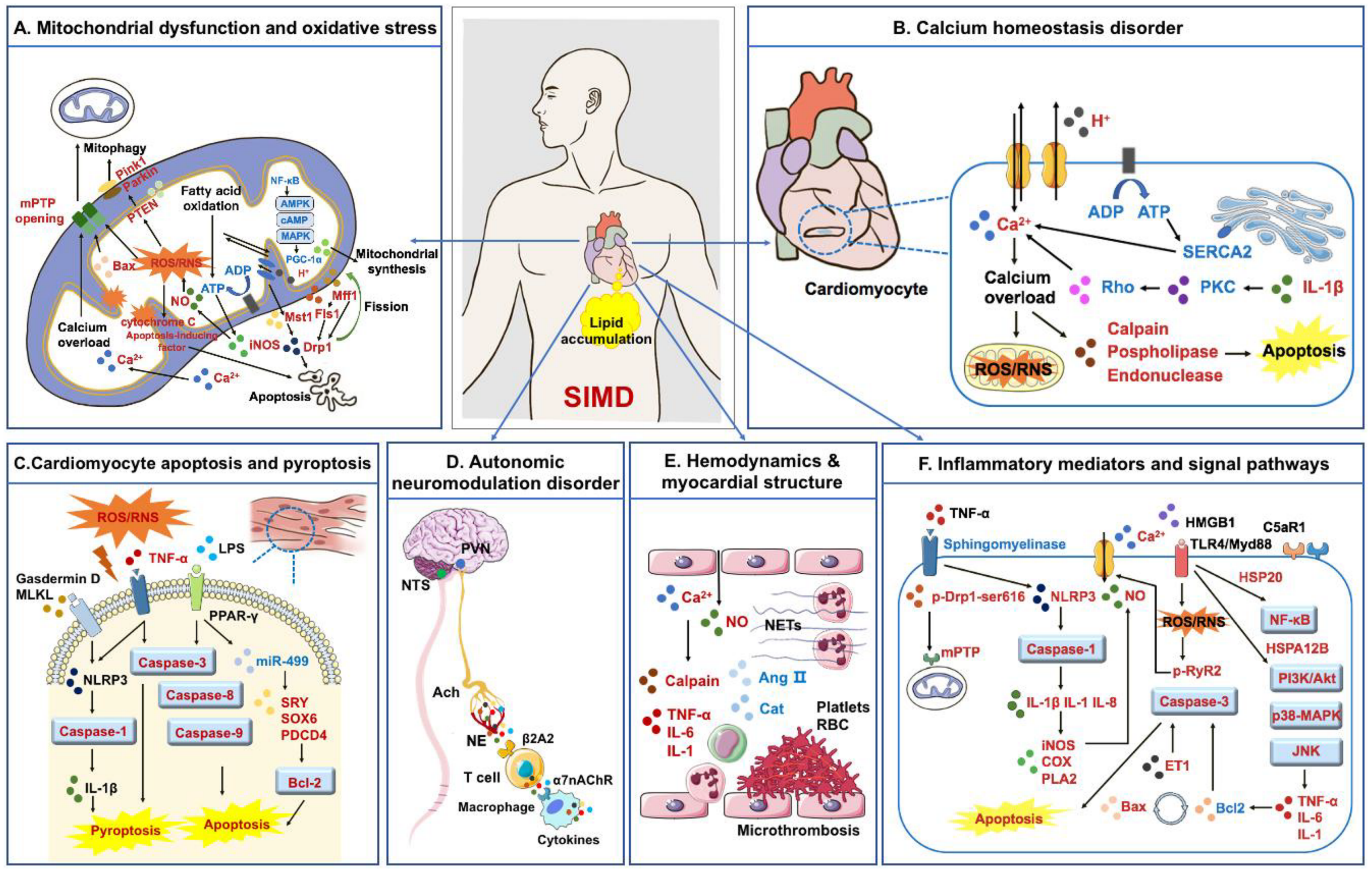
Pathogenesis of SIMD. **(A)** Mitochondrial fission and synthesis disorders, impaired mitochondrial autophagy function, and an imbalance between oxidative stress and antioxidants collectively contribute to SIMD; **(B)** calcium homeostasis imbalance is an important pathological process leading to oxidative stress and apoptosis of myocardial cells; **(C)** myocardial cell apoptosis and pyroptosis are direct factors leading to myocardial injury; **(D)** autonomic neuromodulation disorder is involved in the progression of SIMD; **(E)** hemodynamics and myocardial structural changes are specific manifestations of SIMD; **(F)** inflammatory cytokines and signaling pathways participate in various stages of SIMD formation. Factors in red are up-regulated in SIMD, while factors in blue are down-regulated in SIMD.

The pathogenesis of SIMD is complex. This review found that the pathogenesis of SIMD is related to mitochondrial dysfunction, oxidative stress, cardiomyocyte apoptosis and pyroptosis, dysregulation of myocardial calcium homeostasis, myocardial inhibitory factors, disorders of autonomic nerve regulation, hemodynamics and myocardial structural changes (Figure 2).

#### 3.1.1 Mitochondrial dysfunction

##### 3.1.1.1 Mitochondrial fission, synthesis disorders, and structural damage

Mitochondrial fission and fusion are crucial for maintaining mitochondrial homeostasis and structural integrity. Mitochondrial fission is achieved through the coordinated action of various proteins and protein complexes, including the primary factors dynamin-related protein 1 (Drp1), mitochondrial fission 1 protein (Fis1), and mitochondrial fission factor (Mff) (13). Dynamin-related protein 1, a mitochondrial dynamin-related protein that can be assembled into circular oligomers through protein-protein interactions, is further recognized and located in the mitochondrial outer membrane and is involved in the contraction process of mitochondrial fission (14). Mitochondrial fission 1 protein, an outer mitochondrial membrane protein, is found at the mitochondrial constriction site and interacts with Drp1, forming a bidirectional cellular skeleton to facilitate mitochondrial fission contraction (15). Mitochondrial fission factor is a mitochondrial membrane protein that localizes to the mitochondrial constriction site and interacts with Drp1, promoting Drp1 aggregation and mitochondrial constriction. It has been observed that Mff can also form a complex with Fis1 to facilitate the first steps of mitochondrial fission (13). Mitofusins (Mfn1/2) and optic atrophy 1 (Opa1) are involved in mitochondrial inner membrane fusion that maintains normal mitochondrial morphology and promotes mitochondrial fission (15). Inflammation causes an imbalance in the mitochondrial fission and fusion of cardiomyocytes, reducing energy metabolism and cellular activity and leading to myocardial injury.

Studies have reported that lipopolysaccharide (LPS) can promote Drp1 expression in sepsis models by up-regulating Mammalian STE20-like kinase 1 (Mst1), activating mitochondrial fission, and enhancing mitochondria-related apoptotic signaling. Excessive mitochondrial division causes mitochondrial oxidative damage, decreases mitochondrial membrane potential, shifts mitochondrial pro-apoptotic factors to the cytoplasm/nuclei, impairs mitochondrial energy, and activates mitochondrial apoptosis, leading to cardiomyocyte apoptosis. Simultaneously, excessive mitochondrial fission consumes most of the available actin, resulting in the disaggregation of the myocardial cytoskeleton, impairing myocardial cell contraction and reducing cardiac function (16, 17).

Mitochondrial synthesis in cardiomyocytes is mainly regulated by nuclear factors (18). Many transcription factors are activated in cardiomyocytes, promoting mitochondrial synthesis. The peroxisome proliferator-activated receptor gamma coactivator 1-alpha (PGC-1α) is an important transcription factor that can promote mitochondrial synthesis and metabolism. Activation of PGC-1α can be achieved through several pathways, including cyclic adenosine monophosphate (cAMP), AMP-activated protein kinase (AMPK), and mitogen-activated protein kinases (MAPK) signaling.

Cardiomyocytes can promote mitochondrial synthesis by increasing the rate of protein synthesis and transport, which are crucial for mitochondrial synthesis. The extracellular signal-regulated kinase 1/2 (ERK1/2), the mechanistic target of rapamycin (mTOR) and the proteasome pathway are considered to be the major pathways for protein synthesis and transport. Reconstruction of the membrane structure is also necessary for mitochondrial synthesis, and cardiomyocytes can promote this process by increasing the reconstruction of mitochondrial inner membrane structure, including membrane expansion and fusion. Therefore, the main processes of mitochondrial synthesis in cardiomyocytes include transcriptional regulation, protein synthesis and transport, and membrane structure reconstruction. Sepsis can impair these processes, reduce the number and function of mitochondria in cardiomyocytes, and lead to myocardial injury. The expression of PGC-1α was significantly down-regulated in the heart tissue of septic mice, and mitochondrial biosynthesis was inhibited, leading to cardiac energy production disorders and affecting cardiac function (19, 20). The integrity of the mitochondrial membrane during the development of sepsis is damaged by the tyrosine signal transduction pathway, leading to myocardial mitochondrial dysfunction and oxidative stress, and reduced antioxidant defense (21).

##### 3.1.1.2 Mitochondrial autophagy

Autophagy is a highly regulated cellular process through which cells degrade and recycle their own components, such as damaged organelles, misfolded proteins, and other macromolecules. This process is crucial for maintaining cellular homeostasis and responding to various stressors, including nutrient deprivation, oxidative stress, and infection. Autophagy is mediated by the formation of autophagosomes, which engulf cellular debris, and their subsequent fusion with lysosomes, where the contents are broken down and recycled. The activation of autophagy plays a significant role in maintaining cell survival, regulating inflammation, and preventing the accumulation of toxic substances that may lead to cellular dysfunction.

Mitophagy is activated as a compensatory protective mechanism that can effectively remove damaged mitochondria, reduce damage to cardiomyocytes, and promote recovery of mitochondrial and cardiac function (22). Studies on mitophagy have shown that the phosphatase and tensin homolog (PTEN)-induced kinase 1 (PINK1) Parkinson protein 2 (Parkin) pathway is involved in regulating mitophagy and attenuates cardiomyocyte injury, expression of inflammatory factors and cardiomyocyte injury factors (23). In sepsis, factors such as inflammation and oxidative stress cause damage to cardiac mitochondrial, activating the process of mitophagy. Mitochondrial damage leads to the accumulation of reactive oxygen species (ROS) in cells, activating the PINK1 and Parkin signaling pathways (18).

It has been reported that PINK1 can accumulate on the outer membrane of mitochondria and phosphorylate Parkin protein, activating Parkin and translocating it to the surface of damaged mitochondria. Parkin binds to multiple proteins on the outer membrane of mitochondria and induces the formation of a vesicular membrane structure in areas of mitochondrial outer membrane damage. The vesicular membrane can facilitate the fusion of mitochondria with lysosomes, ultimately forming an autophagosome. The autophagosome is a membrane structure that envelops and encloses mitochondria and eventually fuses with lysosomes for degradation. In sepsis, mitochondrial autophagy can reduce cardiomyocyte death. Studies have reported that further damage to mitochondrial autophagy function can lead to myocardial injury.

#### 3.1.2 Oxidative stress

Under normal fatty acid oxidation conditions, 70% of Adenosine triphosphate (ATP) in the myocardium is derived from fatty acid oxidation; the other part is derived from glucose oxidation (24). In the septic state, interleukin-1β (IL-1β) and other inflammatory factors can down-regulate low-density lipoprotein receptor (LDLR) expression in cardiomyocytes. At the same time, reducing LDLR and fatty acid transporter CD36 can inhibit lipid uptake in cardiomyocytes (24, 25). Sepsis inhibits intracellular fatty acid oxidation, ultimately reducing ATP production in cardiomyocytes and leading to myocardial dysfunction.

Mitochondrial oxidative stress occurs in the early stages of sepsis. When the body is infected, inflammatory cells (such as neutrophils and macrophages) can release some inflammatory mediators, such as cytokines and chemokines, which can further activate oxidative stress reactions and increase oxidative stress. At the same time, the hypoxic state and metabolic abnormalities during the infection process can also lead to increased mitochondrial free radical production. Oxidative stress can impair the mitochondrial oxidative phosphorylation system and respiratory chain, leading to mitochondrial dysfunction and disturbances in myocardial energy metabolism and calcium ion homeostasis imbalance. Oxidative stress can also cause the mitochondrial permeability transition, which releases proteins such as cytochrome C and apoptosis-inducing factors from the mitochondria, further inducing apoptosis of myocardial cells. In addition, oxidative stress can oxidatively damage mitochondrial DNA, down-regulating mitochondrial gene expression and synthesis of mitochondrial proteins, ultimately leading to functional and structural abnormalities of myocardial cells. Several studies have confirmed the oxidative-reductive imbalance in sepsis patients (26–30).

Tissue hypoxia inhibits the mitochondrial electron transport chain and oxidative phosphorylation, reduces ATP synthesis, and increases inducible nitric oxide synthase (NOS) expression to produce a significant amount of nitric oxide (NO) (31). It has been reported that a considerable amount of NO can inhibit respiratory chain complexes, producing substantial ROS. Combining NO and superoxide can induce protein dysfunction, lipid peroxidation, and DNA damage. At the same time, NO competes for the oxygen binding site of cytochrome C oxidase, leading to dysfunction of the myocardial mitochondria electron transport chain, resulting in mitochondrial damage and myocardial cell energy failure (32). In addition, by reducing the response of myofibrils to calcium, NO can relax the vascular smooth muscle to alter the pre- and post-cardiac load and downregulate myocardial perfusion β receptor expression, altering adrenal hormone levels and affecting the redox function of mitochondria. Reactive oxygen species can also alter mitochondrial ultrastructure, damage mitochondrial biosynthesis, and affect mitochondrial function through oxidative modification of mitochondrial DNA and other macromolecules, promoting cell apoptosis and aggravating SIMD (33). Inhibition of cytochrome C oxidase disrupts the activity of the mitochondrial electron transport chain enzyme complex (34), so the cell cannot produce ATP through oxygenation, which eventually results in the production of substantial ROS and inhibits the oxidative phosphorylation process (35).

When mitochondrial dysfunction persists, damaged cardiomyocytes produce ROS, and ROS-mediated oxidative stress causes mitochondrial dysfunction, ultimately leading to SIMD (36). When oxidative/antioxidant imbalance occurs, excessive ROS can also directly damage the membrane structure and organelles of cardiomyocytes, thereby increasing the cell membrane permeability, leading to cardiomyocyte dysfunction and even cell autolysis (37). Studies have reported that ROS/reactive nitrogen species (RNS) can cause lipid peroxidation, protein nitrification, and protein oxidation in endothelial cells, leading to abnormal adhesion of leukocytes and platelets and increasing capillary permeability. Endothelial NO synthase (eNOS) is upregulated in endothelial cells, which weakens endothelial cell-dependent vasodilation and aggravates microcirculatory disorders (38). Oxygen free radicals directly attack mitochondrial membrane proteins and nucleotides, disrupting the integrity of the mitochondrial membrane and the activity of biological enzymes, resulting in cell damage and death. Damage to the mitochondrial membrane can lead to oxidative phosphorylation disorders in the respiratory chain, impairing ATP generation and leading to disorders in energy metabolism (39). Functional proteins on the surface of mitochondria are involved in signal transduction, Ca^2+^ regulation and other processes to cause abnormal signal transduction and excitation-contraction coupling disorders in cardiomyocytes (40).

The production and release of a considerable amount of ROS and RNS lead to structural changes of structural proteins at the mitochondrial permeability transition pore (mPTP). The pathological opening of mPTP increases mitochondrial permeability and causes mitochondrial damage (41). Activation of downstream signaling pathways can lead to cardiomyocyte apoptosis and necrosis (42, 43). Opening of mPTP downregulates expression of the mitochondrial complex Ⅱ/V and significantly decreases ATP concentration in cardiomyocytes (44, 45), leading to mitochondrial dysfunction (46, 47). High expression of the pro-apoptotic protein Bax can cause mPTP to open. In contrast, the anti-apoptotic protein Bcl-2 can inhibit the process. Regulation of Bax/Bcl-2 at mPTP opening alters mitochondrial membrane permeability, ultimately leading to cardiomyocyte apoptosis (48).

#### 3.1.3 Cardiomyocyte apoptosis and pyroptosis

When SIMD occurs, the myocardial autophagy mechanism gets activated to reduce its damage. Autophagy has a cytoprotective effect and can adapt to the pathological state of the myocardium (49). This dynamic process allows damaged cellular components, such as organelles and misfolded proteins, to be phagocytosed, packaged, and fused to lysosomes for degradation. Tumor necrosis factor-α (TNF-α) can induce cardiomyocyte death by upregulating Drp1 expression and enhancing mitochondrial translocation, inhibiting the autophagy function of cardiomyocytes, and leading to irreversible damage that accelerates the development of SIMD (50). Inflammatory mediators and oxidative stress can activate the caspase-3 pathway and lead to apoptosis of cardiomyocyte (51, 52). Endotoxin can activate caspase-3, caspase-8, and caspase-9 of cardiomyocytes, which not only induces end-stage nuclear apoptosis, myofilament, and sarcomere disruption in the myocardium but also decreases the contractile response of ventricular myocytes to norepinephrine (53). While caspase-3 inhibitor application can improve endotoxin-induced myocardial systolic dysfunction (54), LPS can inhibit expression of miR-499, and increase expression of sex-determining region Y (SRY), SRY type HMG box (SOX6) and programmed cell death 4 (PDCD4). In addition, studies have reported that LPS activates the Bcl-2 family signaling pathway, resulting in increased expression of pro-apoptotic genes and decreased expression of anti-apoptotic genes. These results suggest that the miR-499-SOX6/PDCD4-Bcl2 signaling pathway is involved in the pathophysiology of SIMD (55).

Pyroptosis is distinct from apoptosis and cell necrosis. Both apoptosis and pyroptosis involve DNA breakage and destruction of the cell membrane, resulting in leakage of cell contents and complete cell death. The difference between the two is that the execution of apoptosis depends on the mixed lineage kinase domain-like protein (MLKL), which selectively leads to cell membrane oligomerization and plasma membrane translocation, resulting in a change in intracellular and extracellular osmotic pressure and cell rupture. However, pyroptosis depends on the Gasdermin D protein, which does not selectively destroy the cell membrane, but instead leads to the release of cell contents, cell flattening, and cell death (56, 57). The NLR Family Pyrin Domain-Containing 3 (NLRP3) inflammasome/caspase-1/IL-1β pathway may be involved to some extent in the occurrence of systemic inflammatory response syndrome (SIRS) (58). Treatment with an NLRP3 inhibitor in septic mice can significantly inhibit NLRP3-mediated inflammasome formation, caspase-1 activation, and IL-1β secretion and has been shown to be protective for the myocardium (59).

#### 3.1.4 Dysregulation of myocardial calcium homeostasis

Cardiac dysfunction caused by a calcium imbalance in sepsis is related to the reduction in L-type calcium channels, inhibition of the sarcoplasmic reticulum calcium pump, abnormal ryanodine receptors, and decreased myofilaments sensitivity to calcium (60). In sepsis, microcirculation disorders associated with myocardial ischemia and hypoxia, on the one hand, directly affect the production of ATP, reduce the activity of the sarcoplasmic/endoplasmic reticulum Ca^2+^ ATPase 2 (SERCA2), accompanied by the decrease in phospholamban (PLB) phosphorylation, and lead to impaired function of the sarcoplasmic reticulum in the uptake of free calcium. On the other hand, dysfunction of the membrane pump, accumulation of intracellular acid metabolites, and increased Na^+^/H^+^ exchange can lead to the opening of voltage-dependent calcium channels in the cell membrane. A large amount of extracellular Ca^2+^ flows inwards. Exceeding the regulatory capacity of the sarcoplasmic reticulum and calcium-binding protein results in intracellular calcium overload (61). When calcium overload occurs, a substantial amount of Ca^2+^ floods into cells and accumulates in mitochondria, resulting in dysfunction of the mitochondrial respiratory, an increase in oxygen free radicals, and lipid peroxidation damage in mitochondria and cardiomyocytes. At the same time, calpain, calcium-dependent phospholipase, calcium-dependent nucleotide endonuclease, and other digestive enzymes are activated, which can also lead to the apoptosis of cardiomyocytes (62).

It has been experimentally demonstrated that PLB mediates the transport of Ca^2+^ between the cytosol and the sarcoplasmic reticulum. Dephosphorylation of PLB can increase calcium pump activity and Ca^2^+ flux, promoting myocardial contraction. Phosphorylation of PLB can inhibit calcium pump activity and reduce the flow of Ca^2+^, thereby promoting myocardial relaxation (63). Excessive leakage of Ca^2+^ from the sarcoplasmic reticulum and influx across the cell membrane can activate calmodulin-dependent proteins and lead to cardiomyocyte death (60).

Decreased sensitivity of cardiac myofilaments to Ca^2+^ prolongs myocardial contraction and ventricular density, thereby impairing the Frank-Starling effect of cardiomyocytes. The reduced calcium sensitivity may be due to the downregulation of protein kinase C (PKC) and Rho kinase expression by inflammatory factors, such as IL-1β (64). In turn, the increase in Ca^2+^ in immune cells of sepsis patients can promote the production of cytokines by LPS-induced monocytes/macrophages, exacerbate inflammatory responses and vascular leakage, and ultimately lead to impaired cardiac function (65).

#### 3.1.5 Myocardial inhibitory factors

##### 3.1.5.1 Inflammatory mediators and signal pathways

Tumor Necrosis Factor-alpha (TNF-α) can induce cardiomyocyte death by enhancing the phosphorylation level of Drp1-ser616 and mitochondrial translocation (50). In addition, TNF-α can activate sphingolipase on the surface of the cardiac membrane, inhibit Ca^2+^ transport in cardiomyocytes, stimulate NO production, activate proteolytic enzymes, degrade troponin and other important related proteins, and weaken cardiac systolic function (66). Activation of NLRP3 and other protein complexes results in increased release of IL-1 and IL-8, decreasing cyclic adenosine monophosphate expression in cardiomyocytes, inhibiting myocardial contractile function, and ultimately causing myocardial dysfunction (67). On the other hand, IL-1β induces transcription of NOS, cyclooxygenase, phospholipase A2, and other genes by activating the neurophospholipid pathway to cause cardiac dysfunction (68). High mobility group box-1 (HMGB1)-Toll-like receptor 4 (TLR4) interaction induces increased intracellular ROS levels, which enhance oxidative stress and phosphorylation of the type 2 ryanodine receptor (RyR2), resulting in Ca^2+^ leakage and attenuating cardiomyocyte contractility (69).

Several studies have reported that the C5a complement is involved in cardiac dysfunction in sepsis, and myocardial injury *in vivo* was prevented by blocking the C5a antibody. This finding demonstrates that reducing C5a receptor levels in cardiomyocytes from sepsis patients is beneficial for treatment (70). In addition, heat shock protein 72 (HSP72), heat shock protein 20 (HSP20), and heat shock protein A12B (HSPA12B) are beneficial in the management of myocardial injury in patients with sepsis. Heat shock protein 72 can reverse cardiac dysfunction in a sepsis model, and HSP20 can inhibit apoptosis by inhibiting nuclear factor-kappa B (NF-κB) activation (44). Heat shock protein A12B can reduce leukocyte infiltration into the myocardium to improve cardiac dysfunction in sepsis by continuously activating phosphoinositide 3-kinase (PI3 K)/protein kinase B (Akt) signaling (71, 72). Myeloid differentiation primary response 88 (MyD88) promotes neutrophil and macrophage recruitment, endothelial cell adhesion, and blood flow blockage, resulting in myocardial injury. Meanwhile, MyD88 antagonistic mice improve cardiac function by reducing cytokine release, neutrophil infiltration, and apoptosis (73).

The main signaling pathways involved in SIMD include TLR4 nuclear factor-kappaB (NF-κB), TLR4 c-Jun N-terminal kinase (JNK), mitogen-activated protein kinases (MAPK), PI3K/AKT, and other signaling pathways. Activation of Toll-like receptors (TLRs) on monocytes and macrophages increases inflammatory cytokines, promoting immune response activation in the myocardium, thereby causing cardiomyocyte injury. Toll-like receptor signaling can be transmitted through different signaling pathways, such as NF-κB and MAPKs, to increase the production of inflammatory cytokines and interferon-induced genes. Its regulation may affect cardiac dysfunction in sepsis (74–78). Activation of the TLR4/JNK signaling pathway promotes increased production of inflammatory factors such as TNF-α and IL-6 and then downregulates Bcl-2 expression, activates caspase-3 cleavage and activation, and promotes apoptosis (54, 79). Activation of p38-MAPK mediates LPS-induced myocardial systolic dysfunction and TNF-α release and indirectly induces IL-1 and IL-6 production, exacerbating cardiomyocyte injury (80). As a ligand, LPS directly activates the PI3K/AKT signaling pathway by binding to the TLR4 receptor, and activation of the PI3K/AKT signaling pathway can inhibit LPS-induced inflammation and apoptosis (81). The PI3K/AKT pathway is involved in regulating inflammatory response during sepsis and plays a crucial role in maintaining the balance of the internal environment of the body and regulating the immune response (82, 83).

##### 3.1.5.2 Β3 adrenaline receptor

In the early stages of cardiac function impairment, β1 and β2 receptors in cardiomyocytes are downregulated and desensitized, while the β3 receptor content is significantly increased. The negative inotropic effect serves to protect the cardiomyocytes. The persistent increase in β3 receptor results in a continuous negative inotropic effect resulting in myocardial inhibition, which deteriorates cardiac function (84).

##### 3.1.5.3 Endothelin

Persistently elevated levels of endothelin-1 are associated with myocardial dysfunction in sepsis (85). In the rat sepsis model, long-term exposure to endothelin precursors can cause decompensated hypertrophy of ventricular myocytes, leading to increased caspase-3 activity in cardiomyocytes and, ultimately, cardiomyocyte apoptosis (86, 87).

##### 3.1.5.4 Renin-angiotensin system (RAS)

Hypotension and hypovolemia caused by bacterial toxins and cytokines in the septic state rapidly activate the myocardial renin-angiotensin system (RAS), which maintains tissue perfusion. However, excessive secretion of angiotensin Ⅱ (AngⅡ) inevitably causes myocardial injury (88).

#### 3.1.6 Autonomic nervous regulation disorders

During the SIMD process, endotoxin can inhibit the cardiac autonomic nerve conduction pathway, induce apoptosis of neuronal and glial cells, and trigger the depolarization current of the sinoatrial node controlled by the cholinergic nerve due to the increase of plasma cytokines, resulting in the loss of heart rate variability (89–91). High concentrations of catecholamines can also decrease heart rate variability and response to endogenous catecholamines. Loss of heart rate variability indicates a high probability of progression to multi-organ failure and a poor prognosis in sepsis (92).

#### 3.1.7 Hemodynamics and myocardial structural changes

When sepsis occurs, factors such as inflammatory mediators and abnormal distribution of intracellular and extracellular Ca^2+^ lead to endothelial dysfunction of the microcirculation vessels and increased capillary permeability, decreasing the volume of microcirculation vessels (93). Overproduction of NO, decreased reactivity of angiotensin Ⅱ, catecholamines, and other vasoconstrictor factors, and inhibition of signal transduction pathways mediate decreased vascular tone, resulting in systemic hypotension and microvascular response (94). Thrombin deposition leads to microthrombi, inflammatory cell migration, and leukocyte adhesion, which increase blood viscosity and lead to microcirculation disorders. The decrease in microcirculation vessel volume, vascular tone, and microcirculation disorder in sepsis leads to reduced myocardial perfusion, affecting the uptake and utilization of oxygen by cardiomyocytes, the systolic and diastolic functions of the heart to varying degrees and ultimately leading to SIMD. At the same time, the myocardial structure is damaged after sepsis, manifested as calcium overload, increased expression of calpain-1, and enzymatic degradation of actin and myosin, inducing myocardial injury and apoptosis and leading to SIMD (95).

### 3.2 The role of miRNAs in SIMD

**Figure 3:**
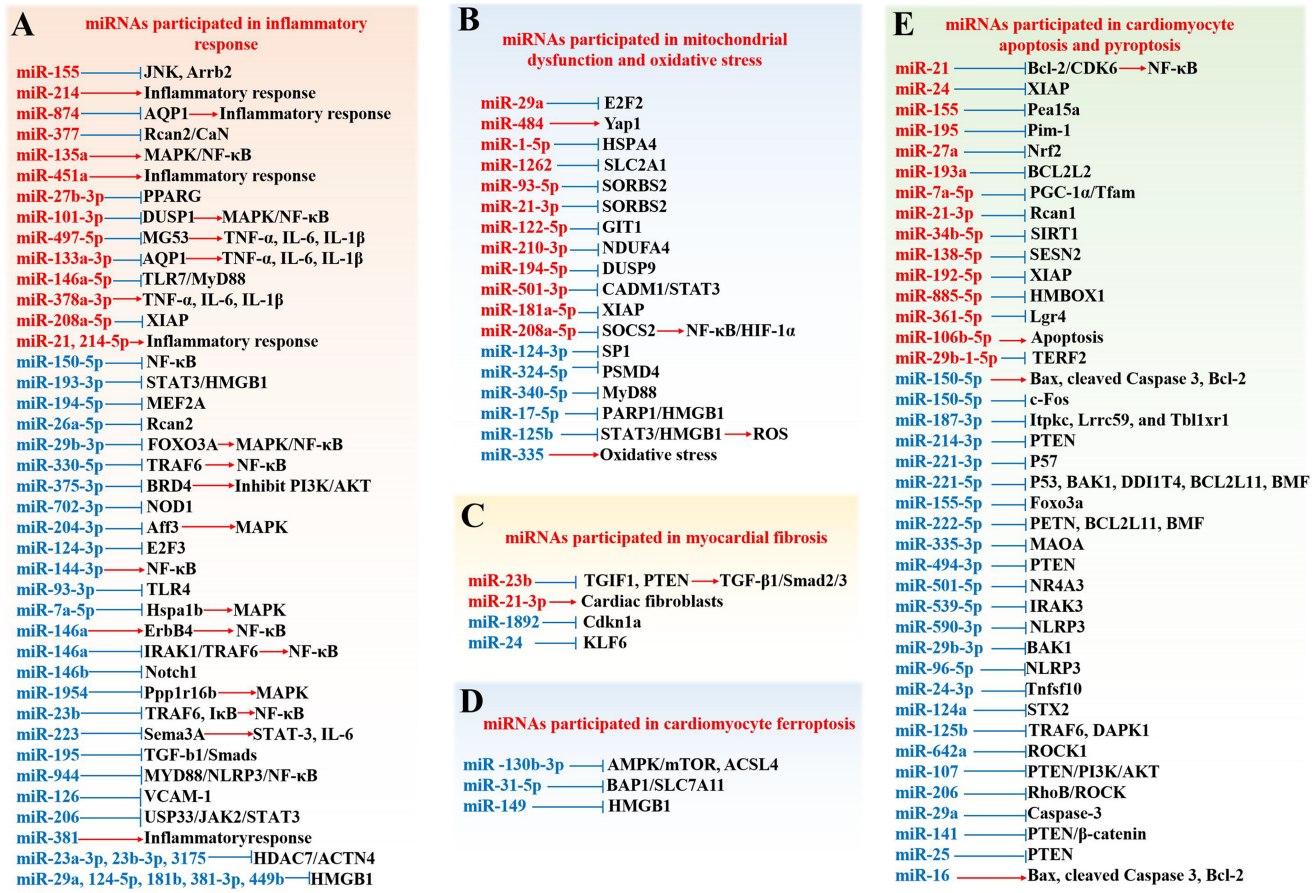
Role and mechanisms of miRNAs in SIMD. **(A)** miRNAs are involved in the inflammatory response; **(B)** miRNAs participate in mitochondrial dysfunction and oxidative stress; **(C)** miRNAs are implicated in myocardial fibrosis **(D)** miRNAs are implicated in cardiomyocyte ferroptosis; E. miRNAs contribute to cardiomyocyte apoptosis and pyroptosis. Factors highlighted in red are up-regulated in SIMD, while those in blue are down-regulated.

At present, the pathogenesis of SIMD is mainly believed to be that after the occurrence of sepsis, the increase of inflammatory reaction and oxidative stress, the disorder of mitochondrial structure and function, and the increase of myocardial cell apoptosis will affect the normal function of the heart. In this process, miRNAs participate in the occurrence and development of SIMD in various ways. As shown in Figure 3, miRNAs are involved in the occurrence, progression, and treatment of SIMD through various mechanisms, including inflammation, mitochondrial function, oxidative stress, cardiomyocyte viability, apoptosis, necroptosis, cardiomyocyte ferroptosis, and myocardial fibrosis. In Figure 3, miRNAs in red font are upregulated in SIMD, indicating that these miRNAs have harmful effects in SIMD. For example, miR-155, miR-214, and miR-874 exacerbate inflammation and contribute to the development of SIMD. Conversely, miRNAs in blue font are downregulated in SIMD, and their upregulation is generally considered beneficial for improving SIMD. For instance, miR-150–5p, miR-193-3p, and miR-194-5p are downregulated in SIMD, and upregulating these miRNAs can suppress inflammation, thereby aiding in the improvement of SIMD.

#### 3.2.1 Inflammatory response

Numerous studies have demonstrated that miRNAs regulate the inflammatory response in SIMD mainly through signaling pathways, including TLR4, MAPK, NF-κB, PI3 K/AKT, JNK, STAT3, and transforming growth factor-β1 (TGF-β1)/Smads and the expression of inflammatory factors such as HMGB1, TNF-α, IL-6, and IL-1β (Table 1). For instance, miR-93-3p can inhibit the TLR4 signaling pathway, protecting against LPS-induced cardiomyocyte inflammation and apoptosis (96). By targeting forkhead box transcription factor 3a (FOXO3A), miR-29b-3p can inhibit the activation of MAPKs and nuclear translocation of NF-κB to block LPS-activated NF-κB signaling, thereby reducing inflammatory injury (97). On the other hand, the overexpression of miR-101-3p/miR-135a can activate the MAPK/NF-κB pathway, inducing SIMD (98, 99). Furthermore, low expression of miRNAs such as miR-23a-3p (107), miR-23b (106), miR-330-5p (100), and others plays a role in the activation of the NF-κB signaling pathway, thereby inducing SIMD. By targeting the Bromodomain-containing protein 4 (BRD4), miR-375-3p can activate the PI3 K/AKT pathway and alleviate SIMD in rats (108). Additionally, miR-155 can target JNK-related inflammatory signals and β-arrestin 2 (ARRB2)-mediated immunosuppression to attenuate cardiac insufficiency and improve survival in late-stage sepsis patients (109). MiR-193-3p (12) and miR-223 (110) can inhibit inflammation and relieve SIMD-related symptoms by targeting the STAT3 signaling pathway. Lastly, miR-195 can activate the TGF-β1/Smads pathway to promote cardiac remodeling and regulate the inflammatory response in septic rats (111).

**Table 1:** Regulation of inflammatory response by miRNAs in SIMD.

Reference	Sepsis model	MiRNA	Expression in sepsis	Target genes	Function
Bi Tang, (96)	*in vitro*	miR-93-3p	**↓**	TLR4	miR-93-3p showed the protective effects against LPS-induced inflammation and apoptosis in cardiomyocytes by inhibiting TLR4 expression.
Zhigang Li, (97)	*in vivo*	miR-29b-3p	**↓**	FOXO3A	miR-29b-3p showed its crucial roles on regulation of apoptosis and production of pro-inflammatory cytokines in NRCMs through directly targeting FOXO3A.
Ye Xin, (98)	*in vivo* + *in vitro*	miR-101-3p	**↑**	DUSP1	Dual specificity phosphatase-1 (DUSP1) was found to be a functional target of miR-101-3p. The downregulation of miR-101-3p led to the overexpression of DUSP1, and the inactivation of the MAPK p38 and NF-κB pathways.
Ge Zheng, (99)	*in vivo*	miR-135a	**↑**	p38mapk/NF-κB	Up-regulation of miR-135a could aggravate sepsis-induced inflammation and myocardial dysfunction via activation of p38 MAPK/NF-κB pathway.
Peng-Cheng Xing, (100)	*in vivo*	miR-330-5p	**↓**	TRAF6	miR-330-5p targeted against TRAF6 to suppress the activation of NF-κB signaling.
Jin Xie, (101)	*in vivo*	miR-146a	**↓**	TLR-4/NF-κB	miR-146a may regulate the TLR-4/NF-κB signaling pathway via negative feedback mechanisms, leading to the improvement of the inflammatory response and cardiac dysfunction in sepsis-induced cardiomyopathy.
Rui An, (102)	*in vitro*	miR-146a	**↓**	ErbB4	Overexpression of miR-146a mitigates myocardial injury by negatively regulating NF-κB activation and inflammatory cytokine production via targeting ErbB4 in LPS-induced sepsis.
Ming Gao, (103)	*in vivo* + *in vitro*	miR-146a	**↓**	IRAK1, TRAF6	miR-146a attenuates sepsis-induced cardiac dysfunction by preventing NF-κB activation, inflammatory cell infiltration, and inflammatory cytokine production via targeting of IRAK and TRAF6 in both cardiomyocytes and inflammatory monocytic cells.
J-L Wei, (104)	*in vitro*	miR-144-3p	**↓**	NF-κB	LncRNA NEAT1 could interact with miR-144-3p to regulate sepsis-induced myocardial cell injury through the NF-κB signaling pathway
Shibo Wei, (105)	*in vitro*	miR-150-5p	**↓**	NF-κB	MALAT1 depletion is responsible for the sepsis inflammatory response by inhibiting the expressions of IL-6 and TNF-α and the NF-κB signaling pathway by upregulating miR-150-5p.
Chao Cao, (106)	*in vivo* + *in vitro*	miR-23b	**↓**	TRAF6, IκB	miR-23b improved sepsis-induced cardiomyopathy by attenuating the inflammatory response, suppressing apoptosis, and preventing NF-κB activation via targeted inhibition of TRAF6 and IκκB.
Qiancheng Luo, (107)	*in vivo*	miR-23a-3p	**↓**	HDAC7/ACTN4	hsa-miR-23a-3p, hsa-miR-3175, and hsa-miR-23b-3p are involved in SIC progression by regulating NF-κB signaling signaling pathway-related HDAC7/ACTN4 in monocytes and cardiac tissue cells.
		miR-3175	**↓**		
		miR-23b-3p	**↓**		
Xiaoyang Hong, (108)	*in vivo*	miR-375-3p	**↓**	BRD4	miR-375-3p targeted BRD4 to activate PI3 K/AKT pathway, thereafter to ameliorate myocardial injury in septic rats.
Yu Zhou, (109)	*in vivo*	miR-155	**↑**	JNK, ARRB2	increased miR-155 expression through systemic administration of miR-155 mimic attenuates cardiac dysfunction and improves late sepsis survival by targeting JNK associated inflammatory signaling and ARRB2 mediated immunosuppression.
Jianyuan Pan, (12)	*in vivo*	miR-193-3p	**↓**	STAT3/HMGB1	miR-193-3p targets STAT3 expression to reduce HMGB1 expression, thereby reducing septic myocardial damage.
Xiaohong Wang, (110)	*in vivo*	miR-223	**↓**	Sema3A	miR-223 negatively regulated the expression of STAT-3 and IL-6 in mouse hearts. loss of miR-223/-223* causes an aggravation of sepsis-induced inflammation, myocardial dysfunction and mortality.
Pengcheng Zheng, (111)	*in vivo*	miR-195	**↓**	TGF- b1/Smads	The MicroRNA-195 and TGF-β1/Smads may promote cardiac remodeling in sepsis rats by up-regulating the nanoantibiotics signaling transduction pathway, thereby having objective curative effect on sepsis rats.
Yuan-Yuan Luo, (112)	*in vivo*	miR-29a	**↓**	HMGB1	Expression of HMGB1, which was repressed by miR-29a targeting. The effect of PVT1 on M1 macrophage polarization was dependent on targeting miR-29a.
Rongmao Nong, (113)	*in vivo*	miR-124-5p	**↓**	HMGB1	HDAC1 bound to miR-124-5p which directly targeted HMGB1.
Hongfeng Gao, (114)	*in vivo*	miR-449b	**↓**	HMGB1	GAS5 was identified to bind with miR-449b and to elevate HMGB1 expression, thus activating the NF-κB signaling.
Lan Ling, (115)	*in vivo*	miR-181b	**↓**	HMGB1	miR-181b directly targeted HMGB1, and downregulation of HMGB1 reduced inflammatory factors and myocardial injury and inhibited cardiomyocyte apoptosis in sepsis.
Jian Liu, (116)	*in vivo* + *in vitro*	miR-381-3p	**↓**	HMGB1	Overexpression of miR-381-3p could repress the mRNA expression of HMGB1, inhibit the cell apoptosis and inflammatory response, and motivate the viability of sepsis cells.
Qing Wang, (117)	*in vivo*	miR-378a-3p	**↑**	-	miR-378a-3p antagomir also significantly alleviated the inflammatory responseby down-regulating the expression of TNF-a, IL-6, and IL-1β.
Yuru Chu, (118)	*in vivo* + *in vitro*	miR-133a-3p	**↑**	AQP1	miR-133a-3p decreased the expression of AQP1, increased expression of the inflammatory cytokines, TNF-α, IL-6 and IL-1β.
Chun Liu, (119)	*in vitro*	miR-702-3p	**↓**	NOD1	MiR-702-3p played an important role in the pathogenesis of sepsis cardiomyopathy via targeting NOD1
Jiang Li, (120)	*in vivo* + *in vitro*	miR-497-5p	**↑**	MG53	Upregulate MG53 expression in cardiomyocytes.
Sheng Wang, (121)	*in vivo*	miR-146a-5p	**↑**	TLR7→MyD88	An immunotolerance by rapid IRAK-1 protein degradation via TLR7→MyD88 signaling and proteasome activation,
Yu Fang, (122)	*in vivo* + *in vitro*	miR-874	**↑**	AQP1	H19 acted as AQP1 ceRNA in regulating miR-874 and restoring LPS dysregulated inflammatory responses and myocardial dysfunction.
Xinhua Wang, (123)	*in vivo*	miR-146b	**↓**	Notch1	miR-146b targets to Notch1 and protected cardiomyocytes against inflammation and apoptosis.
Chen Ge, (124)	*in vivo*	miR-214	**↑**	–	miR-214 has a protective effect in SIMI and thereby may provide a potential novel approach to treat SIMI.
Heng Wang, (125)	*in vivo*	miR-451a	**↑**	–	Significantly upregulated expression of miR-451a was found in septic patients with cardiac dysfunction, and the knockdown of miR-451a in sepsis rats improved cardiac function and inhibited inflammatory responses.
Yuanyuan Luo, (126)	*in vivo* + *in vitro*	miR-26a-5p	**↓**	Rcan2	MALAT1 silencing elevated miR-26a-5p to ameliorate LPS-induced myocardial injury by reducing Rcan2.
Chao Zhang, (127)	*in vivo* + *in vitro*	miR-194-5p	**↓**	MEF2A	MIR155HG upregulated MEF2A through interaction with miR-194-5p
Jiawei Xie, (128)	*in vivo*	miR-7a-5p	**↓**	Hspa1b	Participated in protein processing in the endoplasmic reticulum, antigen processing and presentation, and the mitogen-activated protein kinase signaling pathway.
		miR-204-3p	**↓**	Aff3	
		miR-1954	**↓**	Ppp1r16b	
Haiyan Zhao, (129)	*in vivo* + *in vitro*	miR-27b-3p	**↑**	PPARG	miR-27b-3p targeted PPARG and restrained its expression
Chunyan Li, (130)	*in vivo* + *in vitro*	miR-214-5p	**↑**	–	Downregulation of miRNA-214-5p improved sepsis-associated cardiac dysfunction and inhibited inflammatory factors.
Yong Chen, (131)	*in vivo*	miR-381	**↓**	–	overexpression of miR-381 exerted a cardiac protective effect
Jin Peng, (132)	*in vivo* + *in vitro*	miR-21	**↑**	–	aloin exerts protective effects in sepsis-related myocardial damage through regulating cardiac cell viability and inflammatory responses via regulating the SNHG1/miR-21 axis.
Shiji Wang, (133)	*in vivo*	miR-377	**↑**	Rcan2/CaN	The Overexpression of miR-377 Aggravates Sepsis-Induced Myocardial Hypertrophy by Binding to Rcan2 and Mediating CaN Activity
Wei Lv, (134)	*in vitro*	miR-944	**↓**	MYD88/NLRP3/NF-κB	Circ_0003907 sponged miR-944 to aggravate LPS-induced AC16 cell dysfunction via activating the MYD88/NLRP3/NF-κB axis during sepsis
Ling-Jun Xu, (135)	*in vitro*	miR-208a-5p	**↑**	XIAP	SP1-stimulated miR-208a-5p aggravates sepsis-induced myocardial injury via targeting XIAP
Yu-E Wang, (136)	*in vivo*	miR-126	**↓**	VCAM-1	The targeted nanosystem successfully delivered miR-126 and 1,8-Cineole to the injured heart tissues and vessels, reducing inflammatory responses and improving cardiac function.
Wei Dong, (137)	*in vitro*	miR-206	**↓**	USP33/JAK2/STAT3	miR-206 alleviates LPS-induced inflammatory injury in cardiomyocytes via directly targeting USP33 to inhibit the JAK2/STAT3 signaling pathway
Weiwei Chen, (138)	*in vivo* + *in vitro*	miR-124-3p	**↓**	E2F3	miR-124-3p was found to downregulate E2F3 expression, induced cell apoptosis and inflammation response.

Notes: **↑**, upregulated in septic myocardium and/or serum; **↓**, downregulated in septic myocardium and/or serum.

These miRNAs, through their involvement in inflammation, not only contribute to the development of myocardial injury in sepsis but also play a crucial role in sepsis-induced lung injury, renal injury, intestinal damage, and brain injury. Research has shown that miR-144-3p and miR-146a-5p activate the Janus kinase (JAK)/transcription (STAT) signaling pathway (139) and promote dendritic cell activation and glycolysis (140) by targeting Caveolin-2 and autophagy-related 7 (ATG7), respectively, accelerating the occurrence of Sepsis-associated acute lung injury (SA-ALI). miR-21 (141, 142) and miR-223 (143) worsen SA-ALI by targeting the TLR4/NF-κB pathway, miR-23a-3p targets the Polo-like kinase 1 (PLK1)/STAT1/STAT3 signaling pathway to promote M1 polarization of macrophages (144), and miR-23b mainly targets zonula occludens-1 (ZO-1) to induce vascular leakage and aggravate SA-ALI (145). MiR-21-3p promotes the progression of Sepsis-associated acute kidney injury (SA-AKI) by regulating the AKT/cyclin-dependent kinase 2 (CDK2)/forkhead box-1 (FOXO1) pathway [Lin, et al., (146)]. MiR-214-5p exacerbates SA-AKI by inhibiting the glucagon-like peptide-1 receptor (GLP-1R)/AMPK axis [Guo, et al., (147)]. MiR-214 targets KLF6 [Zhao, et al., (148)] or PTEN [Sang, et al., (149)] to regulate the AKT/mTOR pathway and participate in the pathological process of septic renal injury. Overexpression of MiR-133a-3p promotes apoptosis of septic intestinal epithelial cells by downregulating transgelin-2 (TAGLN2) (150). MiR-141 induces septic intestinal injury through the TLR4 pathway (151). Upregulation of miR-146b-5p inhibits cell proliferation and migration by targeting Cyclin D2 (Ccnd2) and plays a crucial role in the development of septic intestinal injury (152). MiR-25-5p alleviates LPS-induced inflammation, reactive oxygen species generation, and brain injury by negatively regulating the expression of TXNIP (153). Additionally, miR-146a-5p targets TLR7 to regulate innate immune responses in microglia/astrocytes and the intact brain (154).

#### 3.2.2 Mitochondrial function in cardiomyocytes

Mitochondria are the only organelles responsible for generating ATP in body cells. Therefore, their normal function is very important for maintaining the normal pathophysiological functioning of cells, tissues and organs. Because of its continuous pumping action, the heart requires a large amount of ATP to maintain its normal contractile activity. Therefore, the mitochondria in cardiomyocytes must produce adequate energy in the form of ATP through oxidative phosphorylation. In addition, mitochondria play an important role in maintaining proper cardiac function by regulating calcium homeostasis, ROS and related signal transduction in cardiomyocytes.

As shown in Table 2, miR-484 promotes mitochondrial fission by targeting Yes Associated Protein 1, thereby aggravating LPS-induced cardiomyocyte apoptosis and inflammation (155). MiR-324-5p can target proteasome 26S subunit non-ATPase 4 (PSMD4) and act on mitochondrial fission regulator 1 (Mtfr1) to change mitochondrial morphology and promote cardiomyocyte death (156). CircTLK1 can sponge miR-17-5p, activate the poly(ADP-ribose) polymerase 1 (PARP1)/HMGB1 axis, and aggravate the oxidative damage of mitochondrial genome (mtDNA), resulting in mitochondrial dysfunction and cardiomyocyte apoptosis in sepsis (157). Both miR-21-3p and miR-93-5p target SH3 domain-containing protein 2 (SORBS2), potentially causing mitochondrial ultrastructural damage and autophagy, leading to cardiac dysfunction in SIMD (158, 159). MiR-210-3p targets NADH dehydrogenase (ubiquinone) 1 alpha subcomplex 4 (NDUFA4) causing mitochondrial dysfunction and promoting the pathogenesis of SIMD (160). LPS can upregulate MiR-29a, further damaging mitochondrial function, reducing mitochondrial membrane potential (MMP), generating ROS, and damaging cardiomyocytes (162). In sepsis, serum exosomes from patients with SIMD can target the transcription of glucose transporter GLUT1 (SLC2A1) in cardiomyocytes through miR-1262, inhibiting mitochondrial glycolysis rate and promoting cardiomyocyte apoptosis, ultimately aggravating myocardial injury (163). LPS upregulates miR-208a-5p in sepsis, which has been shown to activate the NF-κB/HIF-1α axis mediated by SOCS2, causing mitochondrial swelling and ultimately leading to cardiomyocyte apoptosis (164). MiR-194-5p targets DUSP9 and is involved in oxidative stress and mitochondrial dysfunction, exacerbating sepsis-induced cardiomyopathy (165). In addition, studies have shown that the lncRNA RMRP can regulate post-transcriptional function, inhibit mitochondrial damage, and attenuate cardiomyocyte apoptosis in SIMD by inhibiting miR-1-5p from targeting HSPA4 (161). lncRNA MCM3AP-AS1 alleviates sepsis-induced cardiomyopathy by improving inflammation, oxidative stress, and mitochondrial function through the inhibition of the miR-501-3p/CADM1/STAT3 axis (166).

**Table 2:** miRNAs regulate mitochondrial function in cardiomyocytes.

Reference	Sepsis model	MiRNA	Expression in sepsis	Target genes	Function
Ming Xu, (155)	*in vitro*	miR-484	**↑**	Yap1	miR-484 directly targeted mRNA of Yap1 to inhibit cell viability, and promote apoptosis and inflammation in LPS-treated H9c2 cells.
Jingru Li, (156)	*in vivo*	miR-324-5p	**↓**	PSMD4	PSMD4 and Thumpd3-as1/miR-324-5p/PSMD4 ceRNA network may be important biomarkers and potential therapeutic targets in SCM.
Yu Qiu, (157)	*in vivo* + *in vitro*	miR-17-5p	**↓**	PARP1/HMGB1	CircTLK1 sponged miR-17-5p to aggravate mtDNA oxidative damage, mitochondrial dysfunction and cardiomyocyte apoptosis via activating PARP1/HMGB1 axis during sepsis
Hui Wang, (158)	*in vivo*	miR-21-3p	**↑**	SORBS2	miR-21-3p controls sepsis-associated cardiac dysfunction via regulating SORBS2. Inhibition of miR-21-3p might be a protective strategy to treat sepsis-induced cardiac dysfunction.
Bin Shan, (159)	*in vitro*	miR-93-5p	**↑**	SORBS2	H19 sponged miR-93-5p to promote SORBS2 expression.H19 suppressed sepsis-induced myocardial injury via regulation of the miR-93-5p/SORBS2 axis.
Dandan Chen, (160)	*in vivo* + *in vitro*	miR-210-3p	**↑**	NDUFA4	miR-210-3p promoted SIMD pathogenesis by targeting NDUFA4 to enhance cardiomyocyte apoptosis and impair mitochondrial function.
Ying Han, (161)	*in vivo* + *in vitro*	miR-1-5p	**↑**	HSPA4	RMRP inhibits LPS-induced apoptosis of cardiomyocytes and mitochondrial damage by suppressing the post-transcriptional regulatory function of miR-1-5p on HSPA4.
Xinghua Pe, (162)	*in vivo* + *in vitro*	miR-29a	**↑**	E2F2	TTN-AS1 regulated the expression of E2F2 by targeting miR-29a, abrogated the suppressive effect of TTN-AS1 overexpression on myocardial injury.
Fangyuan Sun, (163)	*in vitro*	miR-1262	**↑**	SLC2A1	Exosomes derived from patients with sepsis may inhibit glycolysis and promote the apoptosis of human myocardial cells through exosomal hsa-miR-1262 via its target SLC2A1
Haichun Ouyang, (164)	*in vivo*	miR-208a-5p	**↑**	SOCS2	high expression of SOCS2 or inhibition of miR-208a-5p alleviates the myocardial injury of sepsis mice via modulating NF-κB/HIF-1α pathway
Jie Wang, (165)	*in vivo*	miR-194-5p	**↑**	DUSP9	microRNA-194-5p (miR-194-5p) participates in the regulation of oxidative stress, mitochondrial dysfunction, and apoptosis. miR-194-5p inhibition could mitigate SIC via DUSP9 *in vivo*
Xiangbi Nie, (166)	*in vivo* + *in vitro*	miR-501-3p	**↑**	CADM1/STAT3	MCM3AP-AS1 acted as a competitive endogenous RNA to repress miR-501-3p, enhance CADM1 expression, and dampen STAT3/nuclear factor-kappaB (NF-κB) activation.

Notes: **↑**, upregulated in septic myocardium and/or serum; **↓**, downregulated in septic myocardium and/or serum.

Additionally, studies have shown that these miRNAs can regulate mitochondrial function and contribute to the progression of liver injury in sepsis. Upregulation of miR-155 exacerbates sepsis-induced liver damage by targeting nuclear factor E2-related factor (Nrf-2) and mediating oxidative stress-induced endoplasmic reticulum stress and mitochondrial dysfunction (167). In contrast, the inhibition of miR-155 attenuates sepsis-induced liver injury by enhancing the expression of suppressor of cytokine signaling 1 (SOCS1) and blocking the JAK/STAT pathway (168).

#### 3.2.3 Oxidative stress

Oxidative stress refers to the imbalance between oxidation and antioxidation in the intracellular and extracellular environments. As a result, harmful molecules such as oxygen free radicals accumulate, causing oxidative damage. In sepsis patients, the level of oxidative stress is significantly increased due to various factors such as infection and inflammatory reactions. The effect of oxidative stress on cardiac function is multifaceted. First, oxidative stress can cause myocardial cell damage and apoptosis, ultimately leading to a decrease in normal myocardial contractile function and heart failure. Second, oxidative stress can lead to dysfunction of endothelial cells and smooth muscle cells in the cardiovascular system, leading to impaired vascular contraction function and microcirculation disorder, reducing blood supply and oxygen delivery to the heart. Third, oxidative stress can activate inflammation and blood coagulation, exacerbating cardiac load and hypoxia, leading to serious consequences such as myocardial ischemia and myocardial infarction. Therefore, oxidative stress plays an important role in sepsis-induced cardiac dysfunction, and maintaining oxidant/antioxidant balance with antioxidant therapy may be an important strategy for preventing and treating SIMD (Table 3).

**Table 3:** miRNAs regulate oxidative stress levels in cardiomyocytes.

Reference	Sepsis model	MiRNA	Expression in sepsis	Target genes	Function
Mei Wu, (169)	*in vivo* + *in vitro*	miR-124-3p	**↓**	SP1	miR-124-3p improves myocardial injury in septic rats through targeted regulation of SP1 to mediate HDAC4/HIF-1α.
Ying Yu, (170)	*in vivo* + *in vitro*	miR-125b	**↓**	STAT3	miR-125b directly targeted STAT3 mRNA and STAT3 bound with HMGB1 promoter. Overexpression of miR-125b mitigated myocardial dysfunction induced by CLP *in vivo*.
Cong Zhang, (171)	*in vivo*	miR-340-5p	**↓**	MyD88	miR-340-5p overexpression partially alleviated impairment of cardiac function, and oxidative stress injury
Xian Long, (172)	*in vivo*	miR-335	**↓**	–	miR-335 expression was upregulated and an elevation in inflammatory factors and cTNI, BNP, CK, LDH and AST was observed.
Wenliang Song, (173)	*in vivo* + *in vitro*	miR-122-5p	**↑**	GIT1	GIT1 as a target of miR-122-5p, inhibition of miR-122-5p may mitigate sepsis-triggered myocardial injury through inhibiting inflammation, oxidative stress and apoptosis via targeting GIT1
Sicong Luo, (174)	*in vitro*	miR-181a-5p	**↑**	XIAP	Up-regulation of miR-181a-5p or silencing of XIAP reversed the inhibitory effects of SNHG1 on inflammation and oxidative stress

Notes: **↑**, upregulated in septic myocardium and/or serum; **↓**, downregulated in septic myocardium and/or serum.

LPS-treated H9C2 cells inhibited the expression of miR-124-3p, upregulated specific protein 1 (SP1), and targeted histone deacetylase 4 (HDAC4)/Hypoxia-inducible factor-1α (HIF-1α) to mediate oxidative stress, causing myocardial injury in septic rats (169). LPS can inhibit the expression of miR-125b, resulting in the upregulation of downstream target gene transcription 3 (STAT3) mRNA, which binds to the promoter of HMGB1, ultimately leading to the generation of ROS, which can cause autophagy and damage to the cardiomyocytes (170). In sepsis, miR-122-5p level is increased, which reduces the activities of antioxidant enzymes such as catalase (CAT), superoxide dismutase (SOD) and glutathione peroxidase (GSH-px), leading to the generation of ROS and oxidative stress in cardiomyocytes. At the same time, the upregulation of TNF-α, IL-6 and IL-1β coupled with the cleavage of caspase-3 leads to cardiomyocyte apoptosis (173). Overexpression of miR-340-5p can reduce oxidative stress-induced injury in cardiomyocytes and improve cardiac dysfunction in sepsis by targeting MyD88 (171). Up-regulation of miR-335 can protect cardiomyocytes against oxidative stress and alleviate myocardial cell injury in sepsis (172). lncRNA SNHG1 shows a protective effect on cardomyocyes in sepsis by targeting miR-181a-5p/X-linked inhibitor of apoptosis (XIAP) axis. This activity inhibits oxidative stress, inflammation, and apoptosis, which enhances cardiomyocyte viability, and finally alleviates myocardial cell injury (174).

#### 3.2.4 Cardiomyocyte viability, apoptosis and pyroptosis

MiRNAs play a role in SIMD pathogenesis through affecting cardiomyocyte viability and regulating various pathways critical to cardiomyocyte survival, differentiation, and proliferation, and promoting cardiomyocyte apoptosis, pyroptosis, and necroptosis (Table 4). Studies have shown that in the rat model of SIMD, miR-21 (175), miR-106b-5p (176), miR-642a (177), and others may affect the viability of cardiomyocytes, suggesting their role in the pathological process of SIMD. MiR-141 loaded into exosomes derived from bone marrow mesenchymal stem cells can inhibit the expression of phosphatase and tensin homolog (PTEN), further enhance the activity of β-catenin, improve myocardial injury, and reduce inflammatory cell infiltration and apoptosis in septic mice (212). Overexpression of miR-539-5p can promote the survival and proliferation of LPS-treated H9c2 cells and inhibit apoptosis by targeting interleukin-1 receptor-associated kinase-3 (IRAK3) (179). MiR-221-3p, miR-221-5p, miR-155-5p, and miR-222-5p can promote the survival of cardiomyocytes by down-regulating three apoptosis-related proteins: brassinosteroid insensitive 1-associated kinase 1 (BAK1), P53, and PTEN (181). Inhibition of miR-214-3p has been implicated involved in the pathogenesis of SIMD, whereas its overexpression can attenuate SIMD by inhibiting the expression of PTEN, inducing myocardial autophagy, and activating AKT/mTOR pathway (187). Downregulation of miR-16 (188), miR-150-5p (191, 192), miR-501-5p (193), and others promotes myocardial injury in sepsis by regulating apoptosis-related proteins such as Bax/Bcl-2 apoptosis family proteins and caspase-3. Upregulation of miR-21 (190), miR-24 (189), miR-7a-5p (196), and others has also been confirmed to promote apoptosis in septic cardiomyocytes. One study found that exosomes derived from sepsis patients’ blood can upregulate miR-885-5p in AC16 cells, downregulate homeobox containing 1 (HMBOX1) and increase IL-1β and IL-18, further promoting pyroptosis of AC16 cells (184). Similarly, the upregulation of miR-138-5p (185) and downregulation of miR-590-3p (186) can cause cardiomyocyte pyroptosis, leading to SIMD. MiR-96-5p targets NLRP3, leading to cardiomyocyte pyroptosis and exacerbating septic cardiomyopathy (211). Additionally, USF2 transcriptionally inhibits miR-206, activating the RhoB/ROCK pathway, which further promotes pyroptosis in septic cardiomyocytes (210).

**Table 4:** miRNAs regulate cardiomyocyte viability, apoptosis, and programmed death.

Reference	Sepsis model	MiRNA	Expression in sepsis	Target genes	Function
Jun Zhang, (175)	*in vivo* + *in vitro*	miR-21	**↑**	–	The protective influence of Hyp against sepsis-induced cardiac dysfunction was attenuated by miR-21 upregulation.
Yujuan Liu, (176)	*in vivo* + *in vitro*	miR-106b-5p	**↑**	–	The overexpression of miR-106b-5p could significantly abolish the effects of PTENP1 on cardiac function and inflammation.
Jing Wang, (177)	*in vivo* + *in vitro*	miR-642a	**↓**	ROCK1	miR-642a directly targets with 3'-UTR of ROCK1.
Bo Song, (178)	*in vivo*	miR-141	**↓**	DAPK1	MiR-141 could decrease inflammatory response and reduce myocardial cell apoptosis by targeting DAPK1, thereby playing the promising protective role in SIC.
Xiaochen Hu, (179)	*in vitro*	miR-539-5p	**↓**	IRAK3	IRAK3 was verified as a target of miR-539-5p. over-expression of miR-539-5p significantly inhibited the inflammation response, promoted viability and proliferation, and suppressed apoptosis of LPS-treated H9c2 cells.
Lin Zhang, (180)	*in vivo* + *in vitro*	miR-107	**↓**	PTEN/PI3 K/AKT	miR-107 was significantly downregulated in Sepsis. miR-107 activates the PI3 K/AKT pathway by inhibiting PTEN, thus attenuating sepsis-induced myocardial injury and LPS-induced cardiomyocyte apoptosis.
Yu Cao, (181)	*in vivo* + *in vitro*	miR-221-3p	**↓**	P57	miRNA-221 clusters play an antiviral role and balance inflammatory response
		miR-221-5p	**↓**	P53, BAK1, DDI1T4, BCL2L11, BMF	miRNA-221-5p attenuates LPS induced cardiomyocyte damage by regulating the NF-κB and JNK pathways
		miR-155-5p	**↓**	Foxo3a	miRNA-155-5p has a double-sided effect on the heart.
		miR-222-5p	**↓**	PETN, BCL2L11, BMF	miRNA-222-5p exerts myocardial protection by inhibiting apoptosis
Yin-Xue Song, (182)	*in vitro*	miR-29a	**↓**	–	GRA exerted an effective role against LPS-induced acute cardiac injury through impeding apoptosis and inflammation regulated by miR-29a.
Wei-Liang Xue, (183)	*in vivo*	miR-27a	**↑**	Nrf2	rhTNFR:Fc activated Nrf2 pathway to protect myocardium against LPS-induced sepsis injury via miR-27a regulation
Guo-Wei Tu, (184)	*in vivo*	miR-885-5p	**↑**	HMBOX1	Sepsis-exos increased the level of miR-885-5p, decreased HMBOX1, elevated IL-1β and IL-18, and promoted pyroptosis in AC16 cells.
Li An, (185)	*in vivo* + *in vitro*	miR-138-5p	**↑**	SESN2	miR-138-5p can negatively mediate SESN2.
Jing-Jing Liu, (186)	*in vivo* + *in vitro*	miR-590-3p	**↓**	NLRP3	ZFAS1, activated by SP1, aggravates the progression of sepsis-induced cardiac dysfunction via targeting miR-590-3p/AMPK/mTOR signaling-mediated autophagy and pyroptosis of cardiomyocytes.
Zhenzhen San, (187)	*in vivo*	miR-214-3p	**↓**	PTEN	miR-214-3p attenuated SIMD through myocardial autophagy inhibition by silencing PTEN expression and activating the AKT/mTOR pathway.
Yan Wang, (188)	*in vivo*	miR-16	**↓**	–	CAIF plays a protective role in sepsis-induced CHF by inhibiting cardiomyocyte apoptosis and inflammation, possibly by regulating miR-16 demethylation.
Ting Chen, (189)	*in vivo* + *in vitro*	miR-24	**↑**	XIAP	miR-24 directly targeted the 3'UTR of XIAP, and suppressed it, and XIAP was modulated indirectly by CYTOR.
Yu Li, (190)	*in vivo* + *in vitro*	miR-21	**↑**	Bcl-2, CDK6	Bcl-2 and CDK6 were found to be the direct target of miR-21 using dual-luciferase reporter and RNA immunoprecipitation assays.
Xiao Geng Zhu, (191)	*in vivo* + *in vitro*	miR-150-5p	**↓**	–	the expression of miR-150-5p was reduced, and overexpression of miR-150-5p with mimics resulted in a decrease in apoptosis, decreased expression of cleaved caspase-3 and Bax, and increased expression of Bcl-2.
Xin Wang, (192)	*in vivo* + *in vitro*	miR-150-5p	**↓**	c-Fos	XIST/miR-150-5p/c-Fos axis affected septic myocardial injury
Lan Gao, (193)	*in vivo*	miR-501-5p	**↓**	NR4A3	miR-501-5p exerted an inhibitory effect on cardiomyocyte apoptosis and inflammation in a NR4A3-dependent manner.
He Ma, (194)	*in vivo*	miR-125b	**↓**	TRAF6	*in vitro* transfection of endothelial cells with miR-125b mimics attenuate LPS-induced ICAM-1 and VCAM-1 expression by suppressing TRAF6 and NF-κB activation.
Hui Xiao, (195)	*in vivo*	miR-29b-3p	**↓**	BAK1	CircPTK2 competitively bound to miR-29b-3p to upregulate BAK1 mRNA level, to promote cardiomyocyte apoptosis, inflammatory response, and myocardial damage of the myocardium of septic mice.
Dongshi Liang, (196)	*in vitro*	miR-7a-5p	**↑**	PGC-1α, Tfam	Down-regulation of xist and mir-7a-5p reduces apoptosis in response to LPS.
Dan-Dan Chen, (197)	*in vivo* + *in vitro*	miR-34b-5p	**↑**	SIRT1	ZFAS1 sponged miR-34b-5p and thus elevated expression of SIRT1, which was prohibited by miR-34b-5p.
Zhimin Zhang, (198)	*in vivo* + *in vitro*	miR-195	**↑**	Pim-1	Overexpression of LUADT1 was found to upregulate the expression of Pim-1, a target of miR-195.
XingCheng Sun, (199)	*in vivo*	miR-24-3p	**↓**	Tnfsf10	Up-regulating miR-24-3p from M2-exo imposes cardioprotection against myocardial injury after sepsis through reducing Tnfsf10 expression.
Hui Wang, (200)	*in vivo*	miR-155	**↑**	Pea15a	Pharmacological inhibition of miR-155 using antagomiR improved cardiac function and suppressed cardiac apoptosis induced by LPS in mice as determined by echocardiography
Fangyuan Sun, (201)	*in vivo* + *in vitro*	miR-192-5p	**↑**	XIAP	Down-regulation of KCNQ1OT1 advances cardiac injury through regulating miR-192-5p/XIAP axis during sepsis.
Peng Wu, (202)	*in vivo*	miR-494-3p	**↓**	PTEN	miR-494-3p regulates PTEN expression, inhibits sepsis-induced myocardial injury and protects the function of cardiomyocytes.
Yulong Yao, (203)	*in vivo* + *in vitro*	miR-25	**↓**	PTEN	miR-25 reduced LPS-induced cardiomyocyte apoptosis by down-regulating PTEN/TLR4/NF-κB axis.
Xiufang Diao, (204)	*in vivo*	miR-124a	**↓**	STX2	miR-124a aggravates LPS-induced cardiac dysfunction and the miR-124a/STX2 pathway might serve as the potential diagnostic and therapeutic targets for septic cardiac dysfunction.
Amin M Ektesabi, (205)	*in vivo*	miR-187-3p	**↓**	ITPKC, LRRC59, TBL1XR1	Decreased inflammatory and apoptotic pathways, while increasing cardiac-specific structural and functional, gene expression.
Lian Liang, (206)	*in vivo* + *in vitro*	miR-193a	**↑**	BCL2L2	Sepsis-induced enrichment of miR-193a significantly inhibited cardiomyocytes proliferation and enhanced apoptosis
Yaqing Jiang, (207)	*in vivo* + *in vitro*	miR-29b-1-5p	**↑**	TERF2	miR-29b-1-5p targetedly inhibits TERF2, thereby enhancing sepsis-induced myocardial injury.
Mingwei Gong, (208)	*in vivo* + *in vitro*	miR-21-3p	**↑**	Rcan1	the miR-21-3p/Rcan1 axis may affect apoptosis of cardiomyocytes in sepsis
Danhui Li, (209)	*in vivo*	miR-361-5p	**↑**	Lgr4	MiR-361-5p could aggravate myocardial injury in LPS-induced septic mice by targeting Lgr4 to inhibit the Wnt axis.
Wei Dong, (210)	*in vivo* + *in vitro*	miR-206	**↓**	RhoB/ROCK	USF2 activates RhoB/ROCK pathway by transcriptional inhibition of miR-206 to promote pyroptosis in septic cardiomyocytes
Xinran Gong, (211)	*in vivo* + *in vitro*	miR-96-5p	**↓**	NLRP3	USP7 upregulated SOX9 expression through deubiquitination, and SOX9 suppressed miR-96-5p expression by binding to the miR-96-5p promoter region, thereby promoting NLRP3 expression and then exacerbating sepsis-induced myocardial injury and cardiomyocyte pyroptosis.
Yongju Pei, (212)	*in vivo*	miR-141	**↓**	PTEN/β-catenin	miR-141 was found to bind to and suppress PTEN expression, which further enhanced the activity of β-catenin.

Notes: **↑**, upregulated in septic myocardium and/or serum; **↓**, downregulated in septic myocardium and/or serum.

#### 3.2.5 Cardiomyocyte ferroptosis

Ferroptosis, on the other hand, is a form of regulated cell death that is distinct from apoptosis, necrosis, and autophagy. It is characterized by the accumulation of lethal levels of lipid peroxides, particularly in the presence of iron, leading to cellular membrane damage and rupture. Ferroptosis is primarily driven by iron-dependent oxidative stress, where excessive reactive oxygen species (ROS) oxidize polyunsaturated fatty acids in cell membranes, resulting in lipid peroxidation. This process is tightly regulated by key enzymes, such as glutathione peroxidase 4 (GPX4), which normally protects cells from lipid peroxidation. When this defense mechanism is overwhelmed or inhibited, ferroptosis occurs. Unlike other forms of cell death, ferroptosis does not involve the classic apoptotic markers, but rather is marked by specific molecular pathways linked to iron metabolism and lipid peroxidation.

Ferroptosis has been reported in cardiac tissues of patients with sepsis, suggesting its potential role in myocardial injury in SIMD. Recent studies have shown that ferroptosis is involved in the development of SIMD. Resveratrol can upregulate miR-149, downregulate HMGB1, and inhibit ferroptosis pathway, thereby ameliorating LPS-induced endotoxemic cardiomyocyte injury (213). MiR-130b-3p mitigates septic cardiomyopathy by combating ferroptosis through the regulation of the AMPK/mTOR signaling pathway and directly targeting ACSL4 (214). Additionally, miR-31-5p alleviates sepsis-induced cardiomyopathy by inhibiting the deubiquitination of SLC7A11 via targeting BAP1, thereby also counteracting ferroptosis (215).

#### 3.2.6 Myocardial fibrosis

MiRNAs also play an important role in SIMD by regulating myocardial fibrosis and ferroptosis pathway (Table 5). Studies have shown that LPS induces the upregulation of plasmacytoma variant translocation 1 (PVT1) in myocardial fibroblasts. In turn, PVT1 inhibits the expression of miR-24 and upregulates Kruppel-like factor 6 (KLF6), ultimately inducing the proliferation and migration of myocardial fibroblasts (219). MiR-21-3p is overexpressed in cardiac fibroblasts and transferred to cardiomyocytes to induce SIMD, which is associated with elevated N-terminal brain natriuretic peptide and cardiac troponin (216). MiR-23b-mediated TGF-β1/Smad2/3 signaling promotes the differentiation of cardiac fibroblasts by inhibiting TGF-beta induced factor homeobox 1 (TGIF1). MiR-23b also promotes the deposition of extracellular matrix by inhibiting AKT/N-Cadherin signal transduction via PTEN, leading to myocardial fibrosis (217).

**Table 5:** Involvement of miRNAs in myocardial fibrosis and myocardial ferroptosis in sepsis.

Reference	Sepsis model	MiRNA	Expression in sepsis	Target genes	Function
Qing Dai, (138)	*in vitro*	miR-24	**↓**	KLF6	And knockdown of PVT1 inhibited cell viability and migration, alleviated inflammation cytokines production of LPS-treated cardiac fibroblasts. PVT1 negatively regulates miR-24 and KLF6 is a direct target of miR-24.
Tie-Ning Zhang, (216)	*in vivo*	miR-21-3p	**↑**	–	miR-21-3p was induce in cardiac fibroblasts and was transferred to cardiomyocytes to induce dysfunction.
Haiju Zhang, (217)	*in vivo*	miR-23b	**↑**	TGIF1, PTEN	MiR-23b mediates the activation of TGF-β1/Smad2/3 signaling to promote the differentiation of cardiac fibroblasts through suppression of 5'TG3'-interacting factor 1 (TGIF1). MiR-23b also induces AKT/N-Cadherin signaling to contribute to the deposition of extracellular matrix by inhibiting phosphatase and tensin homologue (PTEN).
Xiaoli Wang, (213)	*in vivo*	miR-149	**↓**	HMGB1	The upregulation of miR-149 downregulated HMGB1, and inhibited the ferroptosis pathway
Zhen Qi, (214)	*in vivo* + *in vitro*	miR -130b-3p	**↓**	AMPK/mTOR, ACSL4	microRNA-130b-3p Attenuates Septic Cardiomyopathy by Regulating the AMPK/mTOR Signaling Pathways and Directly Targeting ACSL4 against Ferroptosis
Yafeng Liu, (215)	*in vitro*	miR-31-5p	**↓**	BAP1/SLC7A11	MiR-31-5p alleviates septic cardiomyopathy by targeting BAP1 to inhibit SLC7A11 deubiquitination and ferroptosis
Cheng-Wu Gong, (218)	*in vitro*	miR-1892	**↓**	Cdkn1a	mmu-miR-1892-Cdkn1a was identified as key regulatory pair via validation,

Notes: **↑**, upregulated in septic myocardium and/or serum; **↓**, downregulated in septic myocardium and/or serum.

#### 3.2.7 Application of miRNAs in the clinical diagnosis of SIMD

At present, miRNAs are regarded as the most promising biomarkers for differentiating SIMD from other myocardial injuries, primarily due to their stable existence in the body's circulatory system. As a result, numerous researchers have endeavored to screen out miRNAs that exhibit specific changes in the serum of patients with SIMD (Table 6). Studies have demonstrated that miR—497, cardiac troponin—I antigen (cTnI), fatty acid binding protein 3 (FABP3), and glycogen phosphorylase BB (GPBB) are elevated in the plasma of children with SIMD. The combination of these clinical indicators holds high diagnostic value, which is of great significance for the early diagnosis and treatment of pediatric SIMD (220). Moreover, miR—132 and miR—223 are negatively correlated with the activities of heart—type isoenzyme (CK—MB), cTnI, TNF—α, and IL—6 in serum, indicating their combined clinical value for the early diagnosis and prognosis assessment of SIMD (221). Additionally, miR—499a—5p also shows good diagnostic and prognostic value for SIMD (222).

**Table 6:** Application of miRNAs in the clinical diagnosis of SIMD.

Reference	Sepsis model	MiRNA	Expression in sepsis	Target genes	Function
Chengjiao Huang, (220)	*in vivo*	miR-497	**↑**	–	Plasma miRNA-497, cTnI, FABP3, and GPBB levels were increased in pediatric sepsis complicated with myocardial injury
Yanping Li, (221)	*in vivo*	miR-132	**↓**	–	Serum miR-132 and miR-223 were negatively correlated with serum CK-MB, cTnI, TNF-α, and IL-6 (*P* < 0.001).
		miR-223	**↓**		
Chuang Yang, (222)	*in vivo* + *in vitro*	miR-499a-5p	**↓**	EIF4E	miR-499a-5p has a good diagnostic and prognostic value for SIMD by inhibiting EIF4E transcription.
Bingyu Zhang, (223)	*in vivo*	miR-29c-3p	**↑**	–	miR-29c-3p has potential as a biomarker for the diagnosis of sepsis, and inhibition of miR-29c-3p expression in animal models reduced sepsis-induced cardiac dysfunction and inflammatory response.
Bin Sun, (224)	*in vivo*	miR-328	**↑**	–	miR-328 is a diagnostic marker for patients with sepsis, and decreasing the expression level of miR-328 can ameliorate cardiac dysfunction and cardiac inflammation in sepsis.
Hailei Guo, (225)	*in vivo*	miR-495	**↓**	–	Overexpression of miR-495 alleviated sepsis-induced inflammation and cardiac dysfunction.
Jesús Beltrán-García, (226)	Patients	miR-21-3p	↑	SORBS2	Upregulation of myocardial inflammation and downregulation of cardiac function
		miR-155	↑	Pea15a	
		miR-135a	↑	–	
		miR-494-3p	↓	–	
		miR-223	↓	Sema3A, STAT-3, IL-6	
		miR-495	↓	–	
	Mice	miR-223	↑	Sema3A, STAT-3, IL-6	
		miR-495	↑	–	
		miR-125b	↑	ICAM-1, VCAM-1, p53, Bax, and Bak1	
		miR-146	↓	IRAK, TRAF6	
		miR-135a	↓	p38 MAPK/NF-kB	
		miR-214	↓	–	
		miR-29b-3p	↓	FOXO3A	

Notes: **↑**, upregulated in septic myocardium and/or serum; **↓**, downregulated in septic myocardium and/or serum.

In comparison with other potential biomarkers for SIMD, miRNAs have their unique advantages. Traditional biomarkers like cTnI and CK—MB have relatively low sensitivity and specificity in the early stage of SIMD. In contrast, miRNAs can be detected at an earlier stage and show more specific changes, enabling more accurate diagnosis. When it comes to diagnostic/therapeutic approaches, current methods mainly rely on clinical symptoms and traditional biomarker detection for diagnosis and drug—based therapies for treatment. The use of miRNAs not only provides a new perspective for diagnosis but also holds the potential for targeted therapy, which may offer more effective treatment options in the future.

Furthermore, inhibition of miR—29c—3p expression in animal models can reduce cardiac dysfunction and inflammation caused by sepsis, indicating its potential as a reliable biomarker for the diagnosis of sepsis (223). Reducing the expression level of miR—328 can improve cardiac dysfunction and inflammation in patients with sepsis, suggesting its role as a diagnostic indicator of sepsis (224). MiR—495 and the Sequential Organ Failure Assessment (SOFA) score are good diagnostic indicators for patients with SIMD, and overexpression of miR—495 can reduce inflammation and cardiac dysfunction caused by sepsis (225). In addition, miRNAs such as miR—21—3p, miR—155, miR—135a, miR—494—3p, and miR—223 in sepsis patients and miR—223, miR—495, miR—125b, miR—146, miR—135a, miR—495, miR—214, and miR—29b—3p in sepsis mice were identified as useful diagnostic markers for SIMD (226).

## 4 Outstanding questions and future perspectives

Sepsis is a severe systemic inflammatory response that often results in cardiac dysfunction. Therefore, investigating the pathogenesis of SIMD, including the role of miRNAs in its development, has clinically significant value. MiRNAs have been found to play a critical role in regulating gene expression, protein synthesis, and cellular function, making their study essential for understanding the development and therapeutic targets of SIMD. In addition, as miRNAs have been shown to play a key role in signal transduction, understanding their regulatory function and biological characteristics can help develop more effective prevention and treatment strategies. By studying miRNAs expression profiles and regulatory mechanisms, new therapeutic targets can be identified, leading to the development of more precise treatment methods.

Although significant progress has been made in understanding the relationship between miRNAs and SIMD, there are still some unresolved issues. In terms of the pathogenesis of SIMD, some studies have a relatively small sample size, which may impact the reliability of the results and make it difficult to accurately reflect the overall situation. For example, when investigating the relationship between mitochondrial function and SIMD, some *in vitro* experiments may not fully simulate the complex *in vivo* physiological environment, leading to results that may deviate from actual conditions. Additionally, in the design of certain experiments, some studies have not adequately accounted for confounding factors, which significantly undermines the accuracy of their conclusions. Similarly, research on the involvement of miRNAs in SIMD also faces related limitations. There are differences in the techniques and methods used to measure miRNA expression levels across studies, which may result in inconsistencies in the findings. Moreover, some studies lack sufficient validation experiments when investigating miRNA target genes, making the relationship between miRNAs and their target genes unclear. For example, with regard to miR-132, different studies report varying effects on cardiac function. Our analysis suggests that these discrepancies may be due to differences in experimental models, the timing of measurements, or variations in the study populations. By analyzing these conflicting pieces of evidence, we can gain a more comprehensive understanding of the role of miRNAs in SIMD, providing valuable insights for future research.

Moreover, although miRNAs demonstrate immense potential as potential diagnostic biomarkers and therapeutic targets for SIMD, several challenges remain in the translational process from basic research to clinical application, particularly in key areas such as stability, delivery mechanisms, off-target effects, and long-term safety. The stability of miRNAs *in vivo* is one of the critical factors affecting their clinical applicability. The presence of abundant ribonucleases in the blood can rapidly degrade miRNAs, resulting in a relatively short half-life in the circulatory system. For instance, some miRNAs may be degraded within hours in serum or plasma samples, making accurate detection and utilization of miRNAs difficult. Furthermore, individual variations in the internal environment, such as pH and enzyme activity, can influence the stability of miRNAs, thereby interfering with the consistency of miRNA-based diagnostic accuracy and therapeutic outcomes. Effective delivery of miRNAs represents another key challenge in clinical translation. As nucleic acid molecules, miRNAs have difficulty crossing cell membranes to reach target cells and exert their effects. Commonly used delivery methods include liposomes, viral vectors, and nanoparticles. While liposomes offer good biocompatibility, they suffer from low encapsulation efficiency and susceptibility to clearance by the reticuloendothelial system. Viral vectors, although highly efficient for transfection, may provoke immune responses, posing safety risks. Nanoparticle-based delivery systems face challenges such as complex preparation processes and unclear potential toxicity. For example, while certain nanoparticle-based delivery systems can effectively deliver miRNAs in animal experiments, they may cause unknown long-term toxicity in human applications due to nanoparticle accumulation in the body. MiRNAs typically exert their effects by complementary binding to target genes, but they may also bind to unintended target genes, leading to off-target effects. Such effects can trigger a series of adverse consequences, such as disrupting the physiological functions of normal cells and causing dysfunction in other organ systems. Taking miRNA-based treatment for SIMD as an example, if miRNAs off-target bind to genes unrelated to cardiac function, it could affect the normal expression of these genes, thereby adversely impacting organs such as the liver and kidneys. While bioinformatic predictions and experimental validation methods can reduce the risk of off-target effects to some extent, completely avoiding these effects remains an urgent challenge. Long-term safety of miRNA therapies is an important factor that must be considered in clinical applications. As miRNAs participate in various intracellular regulatory processes, prolonged modulation of miRNA expression could have unforeseen consequences on the body. For instance, sustained inhibition or overexpression of a particular miRNA might lead to metabolic disorders, immune dysfunction, and other issues. Currently, most studies on miRNA-based therapies are still in animal or early clinical trial stages, with a lack of long-term follow-up data to assess their prolonged impact on human health. This limits the widespread clinical application of miRNA therapies.

## 5 Conclusion

In summary, the pathogenesis of SIMD is complex, involving several factors, including mitochondrial dysfunction, oxidative stress, cardiomyocyte apoptosis and pyroptosis, dysregulation of myocardial calcium homeostasis, myocardial inhibitory factors, autonomic nervous regulation disorders, hemodynamic changes, and alterations in myocardial structure. MiRNAs play significant roles in the progression of SIMD by regulating mitochondrial function and oxidative stress levels, myocardial cell vitality, apoptosis and pyroptosis, inflammatory reactions, myocardial fibrosis, and myocardial cell ferroptosis. Certain miRNAs have strong potential for clinical applications as early indicators for the diagnosis of sepsis-related cardiac dysfunction. Furthermore, many of the miRNAs that target SIMD are also involved in the development and treatment of other sepsis-associated injuries in various organs, such as lung, kidney, intestines, liver and brain.

## Data Availability

The original contributions presented in the study are included in the article/Supplementary Material, further inquiries can be directed to the corresponding authors.
